# Potential Synergies of *β*-Hydroxybutyrate and Butyrate on the Modulation of Metabolism, Inflammation, Cognition, and General Health

**DOI:** 10.1155/2018/7195760

**Published:** 2018-04-01

**Authors:** Franco Cavaleri, Emran Bashar

**Affiliations:** Biologic Pharmamedical Research, 688-2397 King George Hwy., White Rock, BC, Canada V4A 7E9

## Abstract

The low-carbohydrate high-fat diet (LCHFD), also known as the ketogenic diet, has cycled in and out of popularity for decades as a therapeutic program to treat metabolic syndrome, weight mismanagement, and drug-resistant disorders as complex as epilepsy, cancer, dementia, and depression. Despite the benefits of this diet, health care professionals still question its safety due to the elevated serum ketones it induces and the limited dietary fiber. To compound the controversy, patient compliance with the program is poor due to the restrictive nature of the diet and symptoms related to energy deficit and gastrointestinal adversity during the introductory and energy substrate transition phase of the diet. The studies presented here demonstrate safety and efficacy of the diet including the scientific support and rationale for the administration of exogenous ketone bodies and ketone sources as a complement to the restrictive dietary protocol or as an alternative to the diet. This review also highlights the synergy provided by exogenous ketone, *β*-hydroxybutyrate (BHB), accompanied by the short chain fatty acid, butyrate (BA) in the context of cellular and physiological outcomes. More work is needed to unveil the molecular mechanisms by which this program provides health benefits.

## 1. Introduction

Many of our cells can use fat in the absence of glucose to eventually generate energy (ATP) from the fatty acid. However, the brain cannot receive oxidizable fats, as an energy source, as they cannot readily cross the blood-brain-barrier. The water soluble, lower molecular weight ketone can cross and provide neurons with a very efficient energy source [1, 2]. Neurons can thrive with ketones as a fuel source; and some believe this to be a superior energy source especially for individuals with genetic predispositions or lifestyle induced disease related to impaired glucose metabolism [3], including cognitive deficits [4, 5].

Dietary fat is said to have played a critical role in the evolution of the human brain since the brain needs a dense calorie load to sustain it energetically but also needs the fat building blocks to support its development [6]. This dogma certainly continues today based on research that shows that docosahexaenoic acid (DHA) and other fats play a crucial role in neural tissue growth and function. Anomalies in fat metabolism or shortfalls in dietary fats can interfere with brain development and function [7]. In fact, experts assert that a move away from the higher fat diet might be more the cause of the North American metabolic syndrome epidemic.

Statistical review of US macronutrient consumption today reveals an increase in the incidence of obesity correlating with the reduction in dietary fat; which results consequently in increased intake of carbohydrate foods [8]. Somewhere in the middle closer to the low carbohydrate end of the diet is a happy healthy medium where processed carbohydrate sources are limited and healthy fat is loaded but a good supply of fresh vegetables (low glycemic index carbohydrate sources) still comprises a significant component of the diet [9]. In particular, it is speculated that a lower overall intake of carbohydrate sources is the healthier way to go with these choices making up, for the major part, those of a lower glycemic load and higher fiber constitution.

It is well understood that dietary restriction in the form of calorie or carbohydrate deprivation is conducive to ketogenesis and serum ketone elevation [10]. The low-carbohydrate high-fat diet (LCHFD) has cycled in and out of popularity for decades as a therapeutic program to treat metabolic syndrome [11]; epilepsy [12]; cognitive deficit [13]; neurological disorders like Alzheimer's disease where its shown to downregulate deleterious amyloid protein [14]; as an activator of uncoupling protein activity for thermogenesis [15]; and weight loss [16]. The ketogenic diet is far from a novel concept. The understanding that this dietary protocol can effectively reduce seizure frequency [17] and help treat even drug-resistant epilepsy [18] was established as far back as the 1920's [19, 20].

This review highlights the benefit of the ketogenic or low-carbohydrate high-fat diet and provides compelling evidence of its safety and efficacy. Scientific support and rationale are also presented for the administration of exogenous ketone body and other ketone sources of varying types as a complement to the restrictive dietary protocol or as an alternative to the diet. We recommended a specific protocol that involves the administration of an exogenous ketone, *β*-hydroxybutyrate (BHB), accompanied by the short chain fatty acid, butyrate (BA). This review highlights the synergy provided by this combination of BHB-BA in the context of cell signaling and inflammatory control and its use as a substrate for ATP generation through the TCA cycle.

## 2. What Is a Ketogenic Diet?

The ketogenic diet is represented by a distinct macronutrient profile: 65–70% fat; 20% protein; and 5–10% carbohydrate. A daily carbohydrate intake that does not exceed 75 grams on average is needed in order to stay in ketosis; a 50 gram maximum is preferred. One cup of cooked rice, for example, will topple the ketogenic state with a 50 gram carbohydrate count, as can a large apple or banana for many with a count as high as 40 grams for each. The protein type selected in the diet can also have a major influence on serum glucose status. Leucine, a ketogenic amino acid that is often abundant in a typical diet can have a significant influence in support of the ketogenic initiative, on insulin sensitivity and serum glucose clearance [21]. Other amino acids such as alanine, cysteine, and glycine are highly gluconeogenic and during starvation or energy deprivation can be readily converted to glucose by the body [22]. Gluconeogenic/glucogenic amino acids also include arginine, serine, and proline. Eaten abundantly as in a protein source, these amino acids can counter the ketogenic initiative.

Although there are significant health benefits associated with moderate hyperketonemia whether used for therapy or simply life quality improvement, this state is not easy to attain and maintain without significant planning and dietary sacrifice [23, 24]. In fact, the ketogenic lifestyle is very difficult to maintain today for most of the world's population considering our carb-centered cultures. Carbohydrate sources are also hidden in many processed foods. The controversy generated by the medical community related to ketoacidosis risks just helps the layman public make a choice down the path of least resistance—along the carb-laden course. Nevertheless, nutritional ketosis is very different from ketoacidosis. Ketosis induced by dietary modifications that limit carbohydrate intake (or reduce total calories) and elevate healthy fat intake is very different from that of pathological ketoacidosis associated with type-1 diabetes and related diabetic conditions [25, 26]. Safe hyperketonemia can reach levels as high as 10 mM with extended periods of fasting or persistence with the ketogenic diet [27, 28]. Nevertheless, ketosis tends to be regulated at that point by feedback mechanisms [29]. Ketoacidosis is more characterized by serum ketone levels in excess of 18 mM [30].

  Why a ketogenic lifestyle?

We have become desensitized to the fact that serum glucose, itself, is very toxic to the body when uncontrolled, and we fail to appreciate the parallels with serum ketone bodies. When serum glucose is mismanaged, the resulting advanced glycosylated end products (AGEs) [31, 32] and inflammation [33, 34] pose significant toxicity [35] and risk of morbidity [36]. AGE-modified LDL (low-density lipoprotein) is a driver of atherosclerosis and other cardiovascular diseases [37]. The body's response to a diet that persists to deliver high glycemic index foods is not health promoting. A basic understanding of endocrine physiology tells us that every time we spike insulin due to intake of high glycemic index carbohydrate foods, lipolysis is inhibited and energy substrates are shuttled to storage including to fat cells cells [38]. This activity is conducive to adipose fat accumulation and reduces the potential for fatty acid oxidation as an energy substrate in the sedentary or work-loaded situation.

Serum ketone uptake by cells occurs through pathways independent of insulin [39]. Therefore, as much as insulin resistance can impair glucose availability to insulin dependent cells, the ketone can be utilised as an energy substrate despite the insulin dysfunction. Additionally, evidence shows that elevated serum ketone bodies suppress hepatic glucose output and by this mechanism also help mitigate elevated serum glucose [40]. The ketogenic diet is certainly antidiabetic for the type 2 diabetic patient and can play a role in treatment [41].

What the research indicates is becoming well accepted; carbohydrate restriction can contribute significantly to improvement in weight management [42, 43], enhanced serum glucose management in prediabetic and diabetic patients [44], and even reduced frequency of insulin requirement in cases of insulin dependent diabetic conditions [45, 46]. Carbohydrate restriction is not the only dietary strategy that combats lifestyle-related morbidity.

As beneficial as it can be for weight management and correction of metabolic syndrome, calorie restriction by carbohydrate restriction is notoriously poorly tolerated unless coupled to a compensatory higher fat intake which contributes to an equicaloric outcome [47]. The LCHF diet contributes to down-shifts in serum glucose including fasting serum glucose and glucose tolerance [48]. If the carbohydrate intake is low enough, serum ketone levels can rise to support energy demands and health in multiple profound ways that research is showing to be convincing [49, 50]. Still, poor tolerability of the low-carbohydrate diet is a challenge leading to lack of compliance, and a pill, for too many of us, seems to be an easier solution.

## 3. Endogenous Ketone Generation

The liver is the primary site of fatty acid *β*-oxidation from serum-derived fatty acids for the generation of ketones that can subsequently serve as energy substrates for the brain [51–53]. In a healthy individual with intact pancreatic *β*-cell function and insulin feedback, serum ketone levels are managed spontaneously [54]. Serum ketones, acetoacetate and acetone, as well as the ketone body *β*-hydroxybutyrate, serve as signaling ligands that downregulate hepatic *β*-oxidation of fat [55] to regulate serum load. There is compelling evidence that goes back decades supporting the existence and efficacy of this feedback system and the meticulous regulation of ketone synthesis at the transcriptional level [27].

How does the serum ketone story relate to the serum glucose model? The story is analogous. In both models, poorly regulated levels of the energy substrate can lead to toxicity, but levels that are spontaneously managed by the healthy physiology are supportive of healthy metabolic performance. In fact, the healthy window for serum glucose is not much different from that of the healthy window for serum ketones. A healthy window for serum ketones in the context of hyperketonemia is documented to be 2.0 mM to 8.0 mM serum ketones [56]. This range is considered mild to moderate hyperketonemia produced as a survival mechanism during prolonged periods of starvation [25, 53]. Basal levels for most healthy individuals range from 0.1 mM to 0.2 mM serum ketone [57]. Serum ketones are readily used as energy substrates if glucose is scarce [58] in most tissues of the body including the heart [59] and brain.

Nevertheless, this restrictive diet can be made more tolerable with the administration of exogenous ketone supplementation that offsets the lag and energy drag associated with delayed endogenous ketone production and serum elevation [60, 61]. Upon initialisation of the ketogenic diet, it can take as long as five days to reach serum ketone levels that can meet ATP demands as an alternative substrate to glucose. These transition days can prove to be difficult leading to cheat days; while maintenance of functional serum ketone levels also depends on adherence to the diet [62, 63]. This exogenous ketone supplement, as opposed to other diabetic drugs, may be the support needed to make the ketogenic diet tolerable.

Alternatively, cheating while on the ketogenic diet simply slows metabolic transition to the ketone serving as a primary energy substrate. At about the third day of glucose deprivation, the brain begins escalating its dependence on ketones as an energy substrate, and by 96 hours, the brain can be using ketones for most of its ATP needs [64]. In fact, ketones can serve as ATP substrates to supply as much as 70% of the brain's energy to meet demand [65, 66]. In cases of dementia, including Alzheimer's disease, elevated serum ketones (ketosis) are showing promise as a viable treatment [67–69], and the underlying mechanism is quite fascinating.

## 4. Exogenous Ketone Administration

Endogenous ketone generation is a normal and healthy survival mechanism that allows for resilience to prolonged periods of starvation [58]. However, in addition to serving as an ATP substrate, these ketones also serve as ligands that regulate cell signaling and behavior [27]. Nevertheless, these benefits are limited to the level of compliance by the individual on the ketogenic diet, and compliance is poor especially in children. Supplementing with exogenous ketones or ketone bodies might be the answer to the compliance challenge that concurrently contributes to conducive pharmacology. Exogenous supplementation of the ketone or proketone (BHB) by oral route is showing promise and is successfully in use today since 1975. BHB can readily convert back and forth to the other ketone bodies such as acetoacetate and downstream to acetone. Acetone and acetoacetate are biological ketones comprising serum ketone load [70].

The ketogenic diet as a therapy poses challenges because it requires extraordinary dedication and sacrifice, while causing the user to endure states of malaise during energy substrate transition. For some, the achievement of ketosis is more difficult than for others based on metabolic, genetic, environmental, social, cultural, and lifestyle factors combined. Acute supplementation with an exogenous ketone source can serve as a bridging energy substrate to offset the energy deficit associated with this metabolic transition while providing a serum ketone source that additionally serves as a signaling ligand. However, it may also play a role independent of dietary commitments due to its signaling.

The high dosing used in research and current commercial supplement programs could be deemed unnecessary. Consumers are using doses as high as 10 grams per serving of BHB accompanied by medium chain triglyceride (MCT) inclusions that serve as substrates for *β*-oxidation and BHB generation. The oral MCT load is notoriously associated with gastrointestinal distress including diarrhea in many users [71–75]. In addition, these common BHB supplements carry with them a sodium load that can reach 1300 mg sodium per serving. These extremely high-therapeutic doses should be administered with dose-related degrees of monitoring by a health care professional.

## 5. Benefits of Exogenous Ketone

Therapeutic applications for exogenous ketones and ketone bodies like BHB have merit in multiple disease models. In vivo study of ketone supplementation (BHB) decreased tumor growth and prolonged subject survival independent of other dietary inclusions; including independence from serum glucose levels [76]. BHB is shown to play a role in mediating NLRP3 inflammasome-induced IL-1*β* and IL-18 in human monocytes [77] to positively influence inflammation. This may play a role in the treatment of autoinflammatory diseases. Therapeutic ketosis supported by an exogenous ketone downregulates seizure onset in epilepsy [78]. BHB also helps improve cardiac health by reducing myocardial glucose uptake and increasing blood flow [79].

In the course of treating brain hypometabolic diseases like Alzheimer's disease (AD), 10–20 grams daily of exogenous ketone supplementation in divided doses are used with success [80]. AD is associated with compromised glucose metabolism of central nervous system neurons correlating with cognitive deficit [81–84]. Since the ketone does not depend on insulin for uptake and can be used by the mitochondria of neurons efficiently, its prevalence can help overcome some of the energy shortfalls in these diseased brains [85]. Serum levels do not have to rise significantly in order for the brain to be served with the alternative substrate, and this could factor into minimising potential risk as in minimising the exogenous dose required for treatment. Hyperketonemia, where systemic plasma ketones increase just beyond common baselines of (0.2 mM) are shown to improve brain ketone status and consequently serve neurons with an alternative energy substrate to glucose [80].

Elevated serum ketones are shown to attenuate apoptosis in neurodegenerative disease by supporting healthier mitochondrial activity and inhibiting apoptotic proteins [65]. Neurodegeneration rising out of toxicity, injury, or ischemia results in oxidative stress. Exogenous ketone administration in murine models safely inhibits generation of reactive oxygen species [86]. The ketogenic diet has been well documented to be an efficacious therapy for the treatment of epilepsy, including drug-resistant epilepsy [87, 88]. However, exogenous ketone administration is also being used to successfully treat nontractable epilepsy [78, 89].

In an experimental model, exogenous ketones have been found to increase athletic as well as cognitive performance in rats [90]. However, the extent to which exogenous ketones can regulate or enhance prolonged endurance type exercise in human still remains unknown [91]. Exogenous ketones and ketone bodies can serve as tremendous health-promoting agents, but as will be further demonstrated, the combination of BHB with its molecularly analogous short chain fatty acid, butyric acid (BA), may serve as a more appropriate technology for most users.

## 6. Safety of Exogenous Ketones in the Food Supply and in Therapy

Various ketone sources exist naturally in our food supply. Dairy milk contains levels ranging from 10 to 631 *μ*M, making dairy products a natural source of *β*-hydroxybutyrate [92, 93]. The United States FDA classifies various forms of *β*-hydroxybutyrate as “Generally recognised as safe (GRAS).” Exogenous ketones (or ketone bodies) are safe, but how much is too much?

Human subjects tested an exogenous ketone supply as 395 mg/kg of a ketone ester with and without an accompanying meal to show serum BHB levels one hour after administration. Serum BHB was lower in FED versus EMPTY (2.1 mM ± 0.2 mM versus 3.1 mM ± 0.1 Mm) stomach administration. These extreme doses convert to 31.6 grams ketone ester for an 80 kg (176 lb) person and were well tolerated [94]. Another human trial utilizes an oral dose of (*R*)-3-hydroxybutyl (*R*)-3-hydroxybutyrate, a monoester of the BHB molecule, quantified at 714 mg/kg. These doses convert to 57.1 grams ketone ester for an 80 kg (176 lb) person. Maximum plasma ketones were achieved within 2 hours (3.30 mM BHB and 1.19 mM acetoacetate). This high dose was administered for five days three times per day, and it too was tolerated [95]. A typical 8-hour fast can result in 0.5 mM serum ketones [95]. Within a period of seven fasting days, total blood ketone levels can reach 5 to 7 mM [25, 95]. A toxicity study in rats receiving 12 and 15 g/kg weight (females and males resp.) also supports the safety of the *β*-hydroxybutyrate dosing [96].

Oral sodium D,L-*β*-hydroxybutyrate (1000 mg/kg body weight per day) has been administered to children as young as two years old with cardiomyopathy and leukodystrophy from acyl-CoA dehydrogenase deficiency. Within one week of starting the treatment, improvement was noticed from complete paralysis to restored neurological function after two years to include walking and improved brain MRI. Two additional children with the same condition unresponsive to typical treatment showed progressive improvement with the same treatment [97]. In infant hyperinsulinemic hypoglycemia, two six month olds were treated and monitored for five and seven months. Four and eight gram doses, respectively, were administered and well tolerated [60]. It should be noted that extreme therapeutic dosing such as these requires medical monitoring.

## 7. Health Benefits of Butyrate

Short chain fatty acids, also known as volatile fatty acids, are those typically produced by the microbial community of the intestine. These fatty acids include most abundantly, butyrate, propionate, and acetate generated as by-products of dietary fiber fermentation by the gut's symbiotic microbes [98]. These gut microbes are recognised as pivotal contributors to health in ways that reach beyond even complex immune system support. It's very clear that commensal bacteria partake in the synthesis of vitamins [99], and they produce an important energy source in the form of the short chain fatty acids [100]. The short chain fatty acids they produce cycle back to regulate and maintain the healthy gut microbe population while altering the luminal environment to void pathogens [101, 102]. Luminal butyrate adversely affects pathogenic bacteria like *Escherichia coli*, *Salmonella* spp., and *Campylobacter* spp. [103].

Nevertheless, butyrate's reach beyond the colon where it is generated extends to improve insulin sensitivity systemically [102]. Orally consumed butyrate is shown to induce GLP-1 secretion [104], a hormone known to support the improvement of glucose tolerance and appetite control. In the brain, it delivers profound effects the mechanisms of which are not always clear. It is shown to stimulate neurogenesis in the ischemic brain via brain derived neurotrophic factor (BDNF) upregulation [105]. It has antidepressant-like effects [106]. Research shows that butyrate fed mice remain lean (despite dietary calorie load) [107]; have increased energy expenditure in the form of body heat generation; and tend to be more physically active [108, 109].

Butyrate has been shown to have a significant preventive influence on cardiovascular health [110, 111]; reduce serum triglycerides by as much as 50% compared to controls [112]; and lower endogenous cholesterol production [112]. Butyrate and acetate are reported to protect against diet-induced obesity [107, 113]. Butyrate administration has been shown to improve appetite and food portion control [114].

Research has further shown that butyrate is a key fuel for epithelial cells of the intestinal tract and that it may improve gut lining integrity [115]. Similar to BHB, butyrate is an inhibitor of histone deacetylases (HDAC) to induce global changes in genetic transcription of genes encoding oxidative stress resistance [116]. HDAC modulation is also associated with retrieval of long-term memory to recognition [117]. This regulation of gene transcription also results in improved protection from free radical damage associated with strained or extreme metabolic conditions (and environmental toxins). This genetic influence by butyrate administration also includes neuroprotection and improved memory recall in AD models, similar to that exhibited by BHB [118].

Butyrate is shown to inhibit NF-kB and increased I-kB levels as a countermeasure for improved long-term inflammatory control [119]. Oral sodium butyrate attenuates experimentally induced colitis [120]. Orally administered butyrate exerts an anti-inflammatory effect and remission in Crohn's disease patients through downregulation of NF-kB and IL-1*β* [121]. Intralumen butyrate has been shown to directly support health of the gastrointestinal lining [103], to exhibit trophic effects on intestinal cell proliferation and improve villi status [122]. In addition, butyrate has been shown to be a potent promoter of intestinal regulatory T-cells [123] establishing yet another immune regulating mechanism that promotes better inflammatory control at the mucosal lining as well as a mechanism for its inhibition of cancer [124].

Butyrate is shown to reduce or inhibit the microbiome population responsible for generating propionic acid [125]. Propionic acid is implicated in Autism Spectrum Disorders (ASD) [126]. It is speculated that butyric acid's downregulatory influence of propionic acid-producing gut bacteria is the mechanism for improved cognitive status [127]. Interestingly 70% of children with autism or ASD have gastrointestinal disorders and altered gene expression in the brain as a function of the resulting short chain fatty acid imbalance [128].

The list of health benefits associated with the oral administration of butyrate and other short chain fatty acids is vast (Table 1) and conducive to the goal desired with BHB administration. Passive absorption of water in the colon depends on short chain fatty acid availability [129–131]. Butyrate has been shown to play a role in healthy peristalsis to help normalize movement in cases of constipation or diarrhea [132, 133]. Butyrate serves to support optimal hydration and optimal bowel elimination function [134]. This pharmacology helps counter the potential adverse events associated with BHB supplementation.

Butyric acid is also abundantly supplied in the diet from dairy sources. In fact, butter is one of the richest butyric acid food sources with a naturally inherent supply of 3-4% of its fat content as butyric acid. One tablespoon of butter typically delivers 14 grams of fat; of which 560 mg is butyric acid. It is easily possible for an individual to consume well in excess of 1000 mg of butyrate in a day from natural sources. However, to do so has an excessive exogenous fat, including an exogenous cholesterol consequence. Nevertheless, for those on carbohydrate-restriction and/or calorie-restrictive diets where dairy, especially butter and creams, might be avoided and fiber could be easily limited, dietary butyric acid intake and synthesis will be compromised. A supplemental source such as that discussed here is of significant value.

Butyrate supplementation also directly compounds the benefits offered by a ketogenic diet and exogenous ketone supplementation in a synergistic way. Butyrate induces FGF21 in serum, liver, and adipocytes, which in turn stimulates fatty acid *β*-oxidation and hepatic ketone production [135, 136]. This is a central feature of the butyrate pharmacology that directly synergises its activity to the ketogenic goal. It serves as an inducing signal for ketosis to contribute additive activity to a lower oral BHB dosing. Butyrate itself, can serve as a direct substrate to undergo *β*-oxidation as well [137].

## 8. The Plausible Benefits of Coupling Butyrate (Short Chain Fatty Acid) with BHB

Butyrate serves as a significant synergistic force for ketosis induction; BHB ligand interactions and pharmacology; and general health, fitness, and performance support. As discussed, an exogenous supply of ketones, such as a BHB salt, will provide an immediate alternative energy (ATP) substrate for the brain during periods of calorie or carbohydrate deprivation. However, concurrent butyrate supplementation in the form of sodium, calcium, or potassium butyrate (or its esters) will prompt the body to induce endogenous ketone synthesis; serve as a ligand to stimulate receptors that the ketone or ketone body will also act on; contribute to the improvement of insulin and general metabolic health; support inflammatory and general immune system health; support neurological health; support gastrointestinal health and integrity; and serve as an energy substrate for ATP generation directly—all in parallel with the benefits that concurrent supplementation of the sister ketone body (BHB) will provide.

The value of this combined system in the context of the ketogenic initiative is sensible. It must be highlighted that the common LCHF diet or ketogenic lifestyle is characterized by compromised carbohydrate intake which consequently results in compromised fiber intake. This ultimately gives rise to a potential deficiency in microbiome health and limitations of the potential short chain fatty acid by-products of the microbiome, including butyric acid. In addition, when we consider the fact that the microbiome is at regular risk of attack by environmental and dietary factors and pharmaceuticals such as antibiotics [138, 139], the supplemental butyric acid in the context described is fitting.

## 9. Butyric Acid Turns the Ketone into a Potential Weight Loss Strategy

Supplementation with the ketone body, BHB has come under much scrutiny in the context of weight loss claims. This is especially so with MCT accompanied BHB supplements. The ketone will carry with it a caloric contribution as will the medium chain triglyceride. High doses therefore add calories to the daily intake that need to be worked off. In addition, research demonstrates that a rise in serum ketones can inhibit lipolysis, not induce it, so the blanket claims for fat loss by ketone supplementation are not at all appropriate [53, 140]. On the other hand, research does support appetite control and improved body mass proportions with BA supplementation [107, 112–114]. BA is also shown to improve markers of cardiovascular health [112]. A careful balance using the ketone as a bridging factor for brain/cognitive health and a signaling ligand beyond that associated with the neuron is a fit in the program. Nevertheless, an activation signal such as that from butyric acid to turn on fatty acid *β*-oxidation and the ‘burning' of fat for energy is critical in this context.

Using exogenous BA with an exogenous BHB source may have tremendous value in a prework program where calorie management is also a concern. Leveraging this technology to serve as an efficient energy source for a fasting workout may compound the potential for fatty acid oxidation as an energy substrate. Acute nutritional ketosis is shown in research to reduce lactate production and improve performance (time-trials) potential in some forms of activity (cycling) [141]. It is shown to prevent muscle wasting (catabolism) and protect the brain and other tissues from oxidative activity [142]. It makes sense to apply the combined BHB and BA as a preworkload strategy especially if a fasting workout is the norm.

## 10. A Closer Look at Subcellular Pharmacology

A number of studies have been reported to map the mechanisms responsible for the pharmacology of the BHB-BA complex in the context of dietary supplements. Research demonstrates that various G-protein-coupled HCA receptors serve as targets for endogenous ketone and ketone body ligands [143]. This receptor family is shown to be classified into multiple subtypes that possess distinct features such as ligand specificity. While BHB serves as an efficient agonist for the HCA_2_ receptor, for example, it is not able to serve as an agonist for other HCA receptors. Both BA and BHB are signaling ligands for various receptors involved in neuroinflammatory control, including the HCA_2_ receptor [144].

Other ligands including other ketones can serve as agonists to alternate HCA receptors but they may not be able to trigger a transduction cascade from the HCA_2_ receptor. HCA receptors can be found in various tissue and cell types including adipose and macrophages [143]. The expression of these receptors can also be induced in immune cells such as macrophages by various cytokines to upregulate the subcellular influence of their ligands. Free fatty acid (FFAR) and HCA receptors could very well be central targets for prevention and treatment of type 2 diabetes, obesity, and inflammation [145]. These fatty acid receptors are emerging targets for the treatment of diabetes regulating cholecystokinin, peptide YY, and leptin, factors intimately involved in the regulation of feeding behavior and nutrient balance. The naturally occurring ligands, BHB and BA, are already effectively modulating these therapeutic targets.

All three HCA receptors are expressed in adipocytes. The HCA_1_ receptor is activated by hydroxypropanoic acid (lactate), for example, while HCA_2_'s agonist is BHB, and HCA_3_ is activated by another *β*-oxidation intermediate [146]. The regulatory influence by these two natural butyrates on transcription factors involved in transcribing cytokines that regulate inflammatory cascade and immune system activity is expected to be intimately associated with NF-kB modulation.

Nuclear factor erythroid 2-related factor 2 (Nrf2) is the primary transcription factor that is responsible for initiating responses to oxidative stress. Studies showed that the ketogenic diet systematically induces Nrf2 through mild oxidative and electrophilic stress [147, 148]. Nrf2 transcribes a series of endogenous antioxidant defense systems. The transcription factor translocates to nucleus and binds the antioxidant response element (ARE) to transcribe cytoprotective genes [149]. Nrf2 transcribes the endogenous antioxidant peptides hemeoxygenase-1, catalase (CAT), superoxide dismutase (SOD), and glutathione peroxidase (GSH/GPx) [150–152] in response to stress as a preservation mechanism. It is more recently being targeted as a chemopreventive target with the intention of stimulating endogenous antioxidant saturation to block the damage by cancer and chemotherapy drugs on healthy host cell DNA [153, 154].

Nrf2 induction or overexpression is shown to heighten cellular defense mechanisms during metabolic stress and convey neuroprotection during toxin-induced mitochondrial stress to the point of reduced lesion development [155, 156]. This cellular protection is also seen in the context of chemotherapy where concurrent Nrf2 induction protects healthy cells [157]. Nrf2 induction protects cells from LPS-induced inflammatory activity and mortality [158]. Nrf2 signaling pathways are showing promise as a counteraction to mitochondrial dysfunction in PD [159]. Nrf2 induction is shown to convey critical defense against elevated serum-glucose-induced oxidative injury to cardiac muscle cells [160].

Interestingly, the diabetic condition is associated with downregulation of Nrf2 activity via ERK, a factor speculated to be a contributor to stress-induced insulin resistance in cardiac cells [161]. Studies show that Nrf2 activation can be used as a therapeutic application to “improve metabolic disorder and relieve renal damage” associated with diabetes [162]. The widespread existence of Nrf2 and its role in cellular protection as a master regulator of antioxidant defense make it a viable target for upregulation against toxicity in most organs and tissues of the body [163, 164].

Hemoxygenase-1 expression, regulated and transcribed by Nrf2, plays an important role in the antioxidant defense mechanism alongside other common endogenous antioxidants to support recovery from injury, toxicity, and hypoxia [165, 166]. Ischemia is a common cause of cell dysfunction and death and is a function of the interruption of blood flow or oxygen availability to tissues resulting in deleterious events. It is known to be central to the pathology of stroke and one of the more common causes of permanent cell and tissue damage in heart disease [167]. Hemeoxygenase-1 induction is highly correlated with protection from ischemia in neurons [168] and heart tissue [169]. Overexpression of glutathione peroxidase is also associated with resistance to myocardial ischemic reperfusion injury [170, 171].

Butyrate has also been shown to activate Nrf2 [172, 173]. The literature already shows signs that treatment with BA or its salts (sodium butyrate) alleviates oxidative stress [174] and will improve catalase activity [175]. Preconditioning with BA administration protects myocardial injury associated with ischemia by inhibiting expression of inflammatory cytokines [174]. It protects the pulmonary artery smooth muscle cells from oxidation associated with hyperoxia [175] and improves metabolism and muscle atrophy associated with aging [176].

## 11. Future Research

Many can benefit from the state of ketosis associated with these oral strategies including individuals looking to achieve better appetite control, fitness, improved mentation and energy, improved stamina, cellular protection, and other health modifying goals. Based on the metaanalysis of the literature, these goals may also involve relief from inflammation; cognitive deficit associated with neurological disease, trauma, ADD, or others; improved gastrointestinal health; and support of recovery from exercise or intensive performance training. More clinical work is needed before we know more accurately how these strategies can be used reliably.

The BHB-BA coupling appears to facilitate subcellular activity that counters Nrf2 downregulation associated with diabetic or other metabolic conditions that may adversely influence Nrf2 status. The pharmacology of BA is supportive and possibly synergistic. We will be conducting more work to investigate this potential. Although the evidence demonstrating the health benefits of BHB and BA as a combination for oral administration is compelling, the continued research to unveil the mechanisms driving this pharmacology will be of value.

## 12. Discussion

Based on the current evidence in the literature, the application of the exogenous ketone (body) appears to be a viable strategy supporting tolerance of the ketogenic diet. The administration of BA on its own has been reported to deliver positive results in support of fitness, weight management, cognition, and performance enhancement with or without dietary restriction. The current research initiative in our lab is designed to further investigate the subcellular influence by BHB and BHB-BA on key immune system cells at serum concentrations we are able to achieve as a result of the minimum recommended BHB-BA dose (as high as 1.67 mM serum BHB).

Exogenous BHB-BA supplementation could be a functional strategy that induces *β*-oxidation and helps raise serum ketone levels indicative of ketosis (>0.2 mmol) with or without having to engage in stringent deprivation of macronutrients and consequential micronutrient limitations. The coadministration of BHB with its related BA molecule could appear to be an efficient way to accomplish this goal using extremely low and safe oral doses. As such, a carbohydrate or calorie-restrictive diet may not be necessary in order for one to benefit from therapeutic value associated with the signaling activity by this exogenous BHB-BA supplementation. However, it is expected that ketogenic dietary practices applied concurrently with the exogenous BHB-BA supplementation will serve the therapeutic objective more effectively especially in the case of cognitive disorders and weight management.

Nevertheless, supplementation with the BHB-BA source will support tolerance of a restrictive dietary protocol; therefore, its application is an especially great fit as a complement to the dietary initiative. A supplement such as this BHB-BA type should be labelled with a common warning statement as such—Consult a physician before use. Do not use if pregnant or breastfeeding. Not recommended for Type I diabetic patients.

## Figures and Tables

**Table 1:** Summary of the health benefits of Butyrate that has been reported through scientific research conducted on in vitro and in vivo models including human subjects.

Health benefits	Research model	Reference
Induces GLP-1 secretion	Intestinal L cells	[104]
Stimulates neurogenesis	Rat	[105]
Antidepressant effect	Rat	[106]
Obesity management	Mouse	[107, 113]
Better insulin sensitivity	Mouse	[108]
Improves cardiac health with lower cholesterol production	Human cell line, human	[111, 112]
Improves gut lining integrity	Human epithelial cell	[115]
Neuroprotection and improved memory recall in Alzheimer's disease	Mouse	[117]
Provides improved long-term inflammatory control, including Crohn's disease	Mouse, human	[120, 121]
Reduces propionic acid-producing microbes	Rat	[126]
Maintains healthy peristalsis	Human	[132, 133]
Stimulates fatty acid *β*-oxidation by inducing FGF21	Human	[135, 136]
